# Molecular epidemiology and phylodynamic analysis of enterovirus 71 in Beijing, China, 2009–2019

**DOI:** 10.1186/s12985-023-02028-9

**Published:** 2023-11-03

**Authors:** Jie Li, Zhichao Liang, Da Huo, Yang Yang, Renqing Li, Lei Jia, Xiaoli Wang, Chun Huang, Quanyi Wang

**Affiliations:** 1 Institute for HIV/AIDS and STD Prevention and Control, Beijing Center for Disease Prevention and Control, No.16, Hepingli middle Road, Dongcheng District, Beijing, 100013 People’s Republic of China; 2 Institute for Infectious Disease and Endemic Disease Control, Beijing Center for Disease Prevention and Control, No.16, Hepingli middle Road, Dongcheng District, Beijing, 100013 People’s Republic of China; 3 Beijing office of center for global Health, Beijing Center for Disease Prevention and Control, No.16, Hepingli middle Road, Dongcheng District, Beijing, 100013 People’s Republic of China

**Keywords:** Hand, Foot and mouth disease, Human enterovirus 71, Epidemiology, Phylogenetics, Bayesian phylodynamics

## Abstract

**Background:**

Enterovirus 71(EV71)-associated hand, foot and mouth disease (HFMD) decreased dramatically in Beijing from 2009 to 2019. This study was to investigate the epidemiological characteristics, evolutionary dynamics, geographic diffusion pathway, and other features of EV71 in Beijing, China.

**Methods:**

We conducted a retrospective study of EV71-associated HFMD and its causative agent in Beijing, China, from 2009 to 2019. Phylogenetic and phylogeographic methods based on the EV71 genome were used to determine the evolution features, origin, and spatiotemporal dynamics. Positive selection sites in the VP1 gene were identified and exhibited in the tertiary structure. Bayesian birth-death skyline model was used to estimate the effective reproductive number (Re).

**Results:**

EV71-associated HFMD decreased greatly in Beijing. From 2009 to 2019, EV71 strains prevalent in Beijing shared high homology in each gene segment and evolved with a rate of 4.99*10^− 3^ substitutions per site per year. The genetic diversity of EV71 first increased and peaked in 2012 and then decreased with fluctuations. The time to the most recent common ancestor (TMRCA) of EV71 in Beijing was estimated around 2003 when the EV71 strains were transmitted to Beijing from east China. Beijing played a crucial role in seeding EV71 to central China as well. Two residues (E145Q/G, A293S) under positive selection were detected from both the VP1 dataset and the P1 dataset. They were embedded within the loop of the VP1 capsid and were exposed externally. Mean Re estimate of EV71 in Beijing was about 1.007.

**Conclusion:**

In recent years, EV71 was not the primary causative agent of HFMD in Beijing. The low Re estimate of EV71 in Beijing implied that strategies for preventing and controlling HFMD were performed effectively. Beijing and east China played a crucial role in disseminating EV71 to other regions in China.

**Supplementary Information:**

The online version contains supplementary material available at 10.1186/s12985-023-02028-9.

## Introduction

Hand, foot and mouth disease (HFMD) is a widespread infectious disease characterized by rashes on the hands, feet, mouth, and occasionally buttocks with or without fever. Children under 5 years of age are the most susceptible population. In the past few decades, HFMD has been reported worldwide. HFMD has a significant disease burden in China and attracted considerable attention. From 2009 to 2019, 21.97 million HFMD cases, resulting in 3,561 deaths, were reported to the national surveillance system in China [1].

HFMD is caused by a human enterovirus, among which human enterovirus 71 (EV71), Coxsackievirus A16 (CVA16), and Coxsackievirus A6 (CVA6) were the most prevalent pathogens [2]. Illness caused by CVA16 and CVA6 infection is usually mild, whereas EV71 is responsible for severe central nervous system complications and even death [2].

According to the VP1 region, EV71 is classified into genotypes A, B, C, D, E, and F. Genotypes B and C are further divided into subgenotypes B1–B5 and C1–C5 [3–5]. Since the first reported detection of EV71 in 1998, subgenotype C4 has been the predominant agent circulating in mainland China.

In recent years, the etiology surveillance system in Beijing, and some other provinces in China found that the positive proportion of EV71-associated HFMD decreased dramatically. This phenomenon has attracted considerable attention. However, limited studies were carried out to investigate the molecular epidemiology characteristics of EV71. In this study, we described the epidemiology features and pathogen spectrum of HFMD. We conducted genetic analyses to investigate the phylogenetic characteristics, geographic diffusion pathway, and phylodynamic features of EV71 strains in Beijing from 2009 to 2019.

## Methods

### HFMD surveillance system in Beijing

Clinicians and hospitals have been required to report clinical cases of HFMD to National Notifiable Infectious Disease Surveillance System within 24 h of diagnosis since 2008 in China [6]. More than 400 hospitals located in Beijing were involved in the surveillance system. The etiological surveillance program was performed in one sentinel hospital in each district of Beijing. First five outpatients diagnosed with HFMD each month in sentinel hospitals were included in this program. Nurses collected throat swabs and samples were sent to CDC to identify the virus.

### Virus identification and molecular genotyping

EV, EV71, CVA16, and CVA6 were identified with real-time RT-PCR Kits (DAAN GENE, Guangzhou, China) according to the manufacturer’s instructions (Supplemental Table 1). As previously described, complete nucleotide sequences of EV71 VP1 genes were amplified using specific primers [7]. PCR products of complete VP1 genes were purified and sequenced using ABI PRISM310 Genetic Analyzer. Human rhabdomyosarcoma (RD) cells were used to isolate EV71 from a throat swab specimen. In brief, RD cells were cultured using MEM with 10% fetal bovine serum until the monolayer RD cell covered 75% of the flask bottom. Then the cell culture medium was removed, and the supernatant of the specimen was inoculated on the RD cells. After 1 h of incubation at 36℃ in the presence of 5% CO2, the specimen supernatant was replaced by MEM with 2% fetal bovine serum. The cytopathic effect of the enterovirus-infected RD cells was observed every day. Cells were collected when covered cells showed a cytopathic effect. The full-length genome sequences of isolated EV71 strains were amplified and sequenced as previously described [8].

### Selection of representative EV71 sequences

For EV71 sequences in this study, we reduced the computational load by choosing one closely related sequence that represented sequences with a pairwise distance smaller than 1% collected at the same time. For EV71 strains in mainland China, genome sequences were obtained from the NCBI website (as of 20 June 2021). Sequences with unknown dates or regions or many bases missing from the ORF, containing many N bases, and sequences not EV71 were excluded. Before analysis, we constructed a neighbor-joining phylogenetic tree and selected representative strains from each tree branch. Representative strains should cover more provinces of China and each cluster of the phylogenetic trees. Altogether, three datasets were prepared. Dataset one comprised 86 EV71 genome sequences from Beijing during 2009–2019 (Supplemental Table 2). Dataset two included 156 EV71 VP1 sequences obtained in Beijing. Dataset three comprised 353 EV71 VP1 sequences which included 76 in this study and 277 sequences from 29 provinces in China (Supplementary Table 3).

### Phylogenetic and phylogeography analysis

Based on TreeTime software, root-to-tip regression analysis was performed to test the temporal signal of the datasets [9]. Phylogenetic analysis was conducted using the maximum likelihood (ML) method with 500 bootstrap replicates using MEGA 11 [10]. The genetic distance between groups was calculated under the Kimura 2-parameter model using the bootstrap method.

We performed a Bayesian evolutionary analysis based on 156 EV71 VP1 in Beijing to estimate the rate of evolution, and TMRCA using Markov chain Monte Carlo (MCMC) runs of 200 million generations with BEAST v1.8.4 [11] under a Bayesian Skygrid demographic model [12]. A general time-reversible (GTR) nucleotide substitution model specifying a gamma distribution as a prior on each relative substitution rate and a relaxed uncorrelated lognormal molecular clock model to infer the timescale of EV71 evolution were used. The Bayesian MCMC output was analyzed using Tracer v1.7.1 [11]. The Effective Sample Size (ESS) values for estimates were larger than 200. A Bayesian discrete phylogeographic approach based on 353 EV71 VP1 sequences was conducted as the above. To describe the process of EV71 dissemination, a Bayesian stochastic search variable selection (BSSVS) procedure was used. Markov jumps approach was used to count the expected number of virus lineage movements. Statistical support was assessed using Bayes factors (BF) [13] and summarized using SpreaD3[14].

### Discrete trait and a bayesian tip-association significance testing (BaTS)

To evaluate phylogenetic correlations between different regions, phylogenetically based Association Index (AI) statistic, Parsimony Score (PS) statistic, and Monophyletic Clade (MC) statistics for discrete-trait were estimated using BaTS v0.9 beta [15]. The AI and PS statistics tested the association between different regions and tree topology. The MC index tested which trait was associated with phylogeny. The observed mean and associated 95% confidence intervals were calculated by analyzing trees sampled during the Bayesian phylogenetic reconstruction. The null mean and associated confidence intervals were generated after randomly distributing the phylogeny traits (100 replicas). P values < 0.05 were considered significant from the three statistics.

### Selective pressure analysis

The MEME method was used to find the key selection pressure sites on the VP1 and P1 coding regions of EV71 in Beijing [14, 16]. The significance level of the P value was set at < 0.05. The SLAC method assessed the ratio of non-synonymous substitution to synonymous substitution (dN/dS) of the different datasets [17].

### Estimation of Re for EV71

Bayesian birth-death skyline model [18] implemented in BEAST v2.5 [19] was used to estimate Re for EV71 [18]. The analyses were based on a previously selected GTR substitution model. We employed log-normal distribution with a mean of 0 and a standard deviation of 1.0 for Re. A log-normal prior with M = 2.4 and S = 0.5 was used for the rate of becoming uninfectious. Sampling probability (ρ) was estimated assuming a prior β (α = 1.0 and β = 999). The origin of the epidemic was estimated using a log normal prior with M = 2.7 and S = 0.5. The MCMC analyses were run for 100 million generations and sampled every 10,000 steps. The Bayesian MCMC output was analyzed using Tracer v1.7.1, with an effective sample size of > 200 for each parameter. We used the bdskytools package in R to plot the BDSKY results.

## Results

### EV71-associated HFMD decreased greatly

The epidemiology and pathogen distribution of HFMD cases in Beijing from 2009 to 2019 is shown in Table 1. The annual number of reported HFMD cases ranging from 17,357 to 47,440 (median: 30,843), with the corresponding annual incidence rate ranging from 80.6 to 220.5 cases/100,000 populations (median: 151.7 cases/100,000 populations). Both the reported case number and the incidence rate have fluctuated with one year high and the following year low (Supplementary Fig. 1). Both fatal case number and severe case number have decreased significantly (Supplementary Fig. 2). There was no HFMD-associated death since 2016. The severe case number dropped from hundreds to seven cases in 2019. Moreover, there was a noticeable increase in the median age of HFMD cases after 2016.

**Table 1:** The epidemiology and pathogen distribution of HFMD cases in Beijing, China, 2009–2019

Year	2009	2010	2011	2012	2013	2014	2015	2016	2017	2018	2019
Case Number	24,264	45,385	30,843	38,528	33,144	47,440	28,677	30,240	19,987	32,670	17,357
Population (/10^4^)	1972	1961.2	2018.4	2069.3	2114.8	2151.6	2170.5	2172.9	2170.7	2154.2	2153.6
Incidence rate (/10^5^)	123.0	231.4	152.8	186.2	156.7	220.5	132.1	139.2	92.1	151.7	80.6
Male (%)	14,826 (61.1)	27,372 (60.3)	18,474 (59.9)	23,079 (59.9)	20,392 (61.5)	27,992 (59.0)	17,042 (59.4)	17,770 (58.8)	11,955 (59.8)	19,460 (59.6)	10,224 (58.9)
Age^a^	2.8 (1.8–3.9)	3.0 (2.0–4.2)	3.1 (2.0–4.2)	3.3 (2.0–4.5)	3.0 (1.6–4.4)	3.3 (2.0–4.6)	3.2 (1.8–4.5)	3.5 (2.1–4.8)	3.6 (2.1–5.2)	3.5 (1.7–5.1)	3.9 (2.3–5.9)
Fatal cases (‰)	4 (0.2)	18 (0.4)	5 (0.2)	4 (0.1)	2 (0.1)	2 (0.0)	1(0.0)	0(0.0)	0(0.0)	0(0.0)	0(0.0)
Severe cases (%)	104 (0.4)	610 (1.3)	278 (0.9)	356 (0.9)	152 (0.5)	111(0.2)	50(0.17)	55(0.18)	32(0.16)	45(0.14)	7(0.04)
Etiology examination (n)	1769	4174	3179	4791	2943	2570	1537	1979	1799	2671	1884
EV Positive sample (n)	1204	2285	1508	2194	1762	1636	1114	1390	1222	2073	1450
EV71 (%)	420(34.9)	950(41.6)	608(40.3)	738(33.6)	458(26.0)	616(37.6)	137(12.3)	334(24.0)	244(20.0)	34(1.6)	16(1.1)
CVA16 (%)	634(52.7)	716(31.3)	666(44.2)	1191(54.3)	519(29.5)	729(44.6)	338(30.3)	624(44.9)	189(15.5)	377(18.2)	613(42.3)
Other EV^d^ (%)	150(12.5)	602(26.3)	223(14.8)	233(10.6)	870(49.4)	290(17.7)	643(57.7)	433(31.2)	788(64.5)	1659(80.0)	821(56.6)
CVA6 (%)	NA	NA	NA	NA	623(35.4)	50(3.1)	446(40.0)	311(22.4)	593(48.5)	1238(59.7)	605(41.7)

Age^a^, presented as median (p25, p75)

Before 2013, EV71 and CVA16 were the two major pathogens of HFMD. EV71 accounted for 41.6% of HFMD cases and was the most common pathogen associated with HFMD in 2010. Then EV71 positive proportion declined with fluctuations. In 2013, around 26% of samples were EV71 positive. After a rebound in 2014, the positive proportion decreased to 12.3% in 2015. In 2018 and 2019, EV71 was responsible for only 1.6% and 1.1% HFMD, respectively (Supplementary Fig. 3).

### Genomic characteristics and genetic diversity

A total of 86 EV71 genome sequences were obtained in this study. The nucleotide homology of the genomes of all EV71 was 91.6 ~ 99.9%. The nucleotide divergence of overall mean distance of VP4, VP2, VP3, VP1, 2A, 2B, 2C, 3A, 3B, 3C and 3D regions among these strains were 0.043%, 0.049%, 0.054%, 0.051%, 0.052%, 0.066%, 0.049%, 0.060%, 0.089%, 0.042% and 0.051%, respectively (Supplementary Table 4). All 86 EV71 belonged to the C4a genotype. ML phylogenetic trees constructed based on each gene of 86 EV71 genomes showed that VP4, VP2, VP3, and VP1 were located in the same clade with EV71 (BrCr, U22521). At the same time, 2A, 2B, 2C, 3AB, 3C, and 3D were mainly clustered with CVA4 (High Point, AY421762), CVA14(G-14, AY421769) and CVA16(G-10, U05876) (Supplemental Fig. 4). According to the topological structure of ML phylogenetic trees constructed above, EV71 strains in Beijing were genetically related, shared high homology.

According to the phylogenetic characteristics and nucleotide differences of the 3D region, global EV71 is divided into 17 evolutionary lineages (A- Q) [18]. In this study, all the 3D coding region sequences clustered with lineage I (Supplemental Fig. 5).

### Evolutionary characteristics and population dynamics

**Fig. 1:**
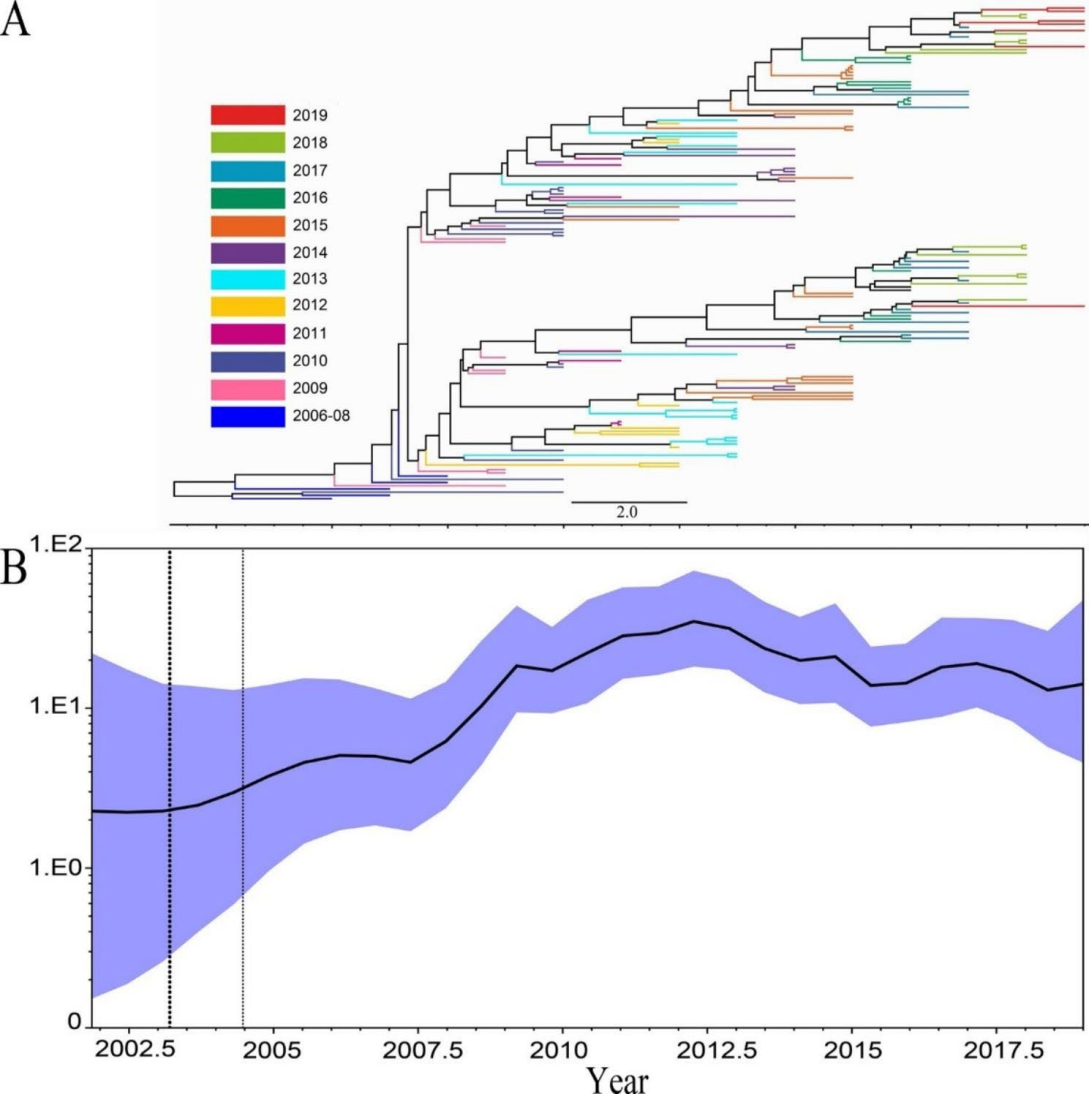
Time-scaled phylogeographic history of EV71 in Beijing from 2009 to 2019. A: The MCC phylogenetic tree was constructed base on the VP1 coding region in Beijing and colored according to the different years. B: Genetic diversity of EV71 VP1 sequences in Beijing. The x-axis represents the year, and the y-axis shows the measure of genetic diversity. The black line shows the median estimates of the EV71 population size, and the blue shading represents the 95% CI.

The evolutionary rate of the VP1 sequence in Beijing was 4.99*10^− 3^ (95%HPD, 4.37–5.59) substitutions/site/year. Via the estimated molecular clock, the common ancestor of genogroup C4a in Beijing was dated to around 2003.20 (95%HPD, 2001.76–2004.47). Maximum clade credibility (MCC) tree constructed using 156 VP1 sequences showed that in around 2006–2007, two clades of VP1 formed on the MCC tree (Fig. 1A). Since then, EV71 were distributed on both clades, young EV71 were located at the end of the branches and presented a ladder-like appearance.

Reconstruction of the demographic history of EV71 in Beijing revealed that since 2007, virus genetic diversity increased greatly and peaked in 2012, then virus diversity decreased from 2012 to 2015 with a slight rebound in 2014. From 2016 to 2019, the virus diversity first experienced an increasing pattern and then a decreasing one (Fig. 1B).

### BaTS and spatial transmission dynamics

The association index (AI) and parsimony score (PS) value with a significance level < 0.001 showed that EV71 VP1 sequences of partial regions were more phylogenetically clustered by region (Supplementary Table 5). The maximum monophyletic clade (MC) of Beijing (p < 0.01), the central (p < 0.01), south (p < 0.01), and west region (p = 0.04) presented high values, which indicated that the geographic structure of EV71 was significant when the strains were grouped by geographical origin.

**Fig. 2:**
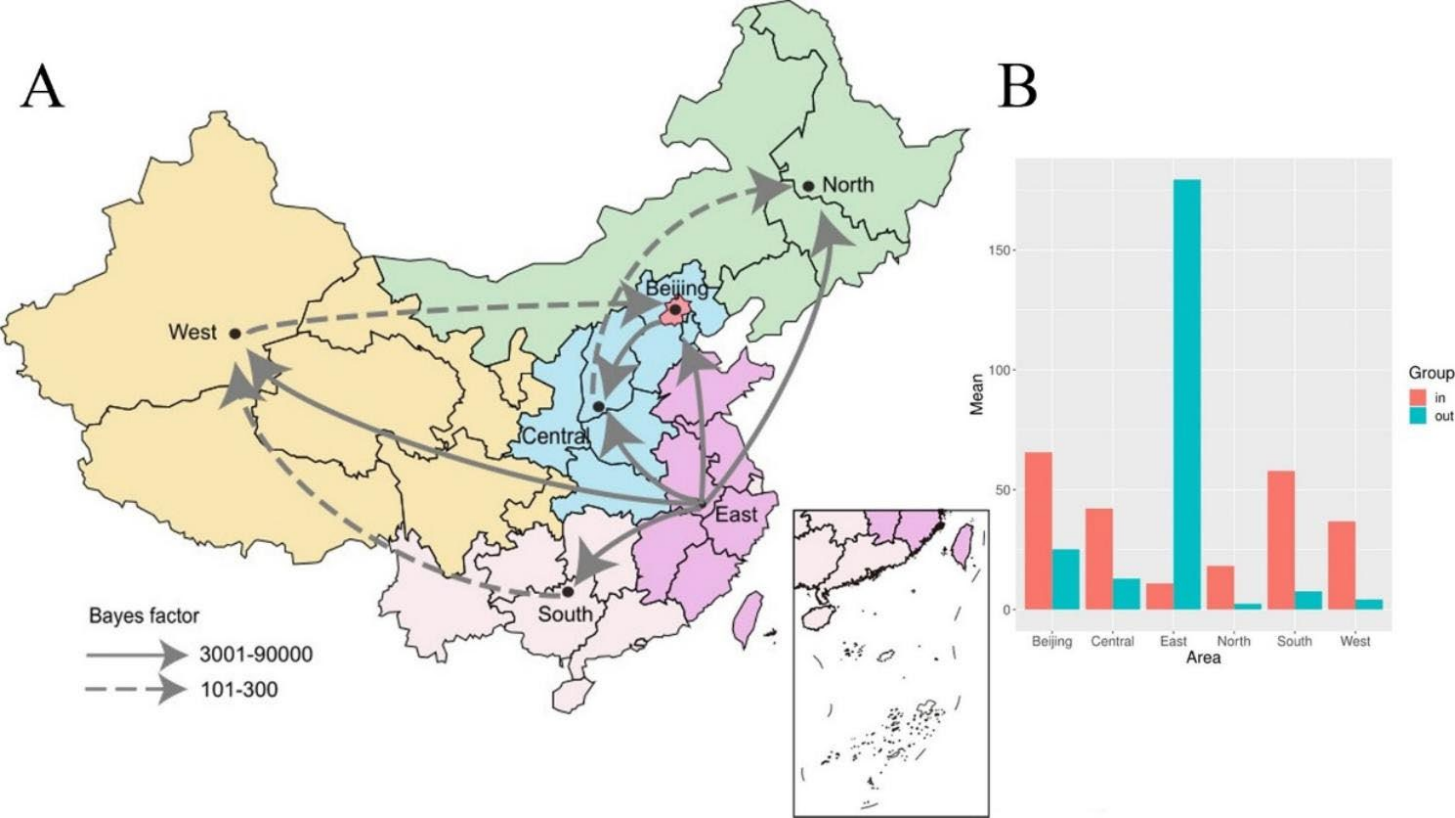
Spatial diffusion of EV71 in China. A: Spatial transmission pathways of EV71 inferred using the Bayesian Stochastic Search Variable Selection method. The solid arrow shows decisive support for the diffusion pathway identified by the VP1 coding regions, with 90,000 > BF > 3,001 and indicator > 0.99. The dashed arrow represents very strong support for the diffusion pathway identified by VP1 coding regions, with 300 > BF > 101 and indicator > 0.98. B: The histogram of the average number of state transitions based on six geographic locations

A past spatial transmission pattern inferred by six regions in mainland China was constructed (Fig. 2A). The result showed that six decisive migration pathways were identified with a very strong BF and posterior probability (PP) support (BF > 3,000 and PP > 0.998). Migrations from east China to Beijing, the south region, the central region, the west region and the north region in mainland China were confirmed through very high BF and PP values. Three strong supportive migration pathways were identified with strong BF and PP (300 > BF > 200 and 0.985 > PP > 0.981) (Table 2). Migrations from Beijing to the central region were confirmed through high BF and PP values. Meanwhile, significant links among the six regions were observed with complicated transmission relations.

**Table 2:** The statistically supported migration rates of EV71 based on VP1 coding regions

From	To	Mean counts	Bayes_factor(BF)	Posterior probability
East	Beijing	54.8228	84535.96413	1
East	South	54.1023	84535.96413	1
East	Central	29.1319	84535.96413	1
East	West	28.1882	84535.96413	1
Beijing	Central	12.8682	16903.77723	0.999747488
East	North	13.1344	3838.468399	0.998888945
Central	North	4.7183	260.7469132	0.983889703
South	West	4.6872	249.6050823	0.983182668
West	Beijing	3.816	220.5715537	0.98101106

Results inferred through the Markov jump method reflected that the east region dominates the out-migration for EV71, which was followed by Beijing. The state counts of inward migration were much larger than that of outward migration in Beijing, the central, south, west, and north regions of mainland China (Fig. 2B). Beijing was the main in-migration area for EV71, followed by the south region.

### Natural selection pressure

**Fig. 3:**
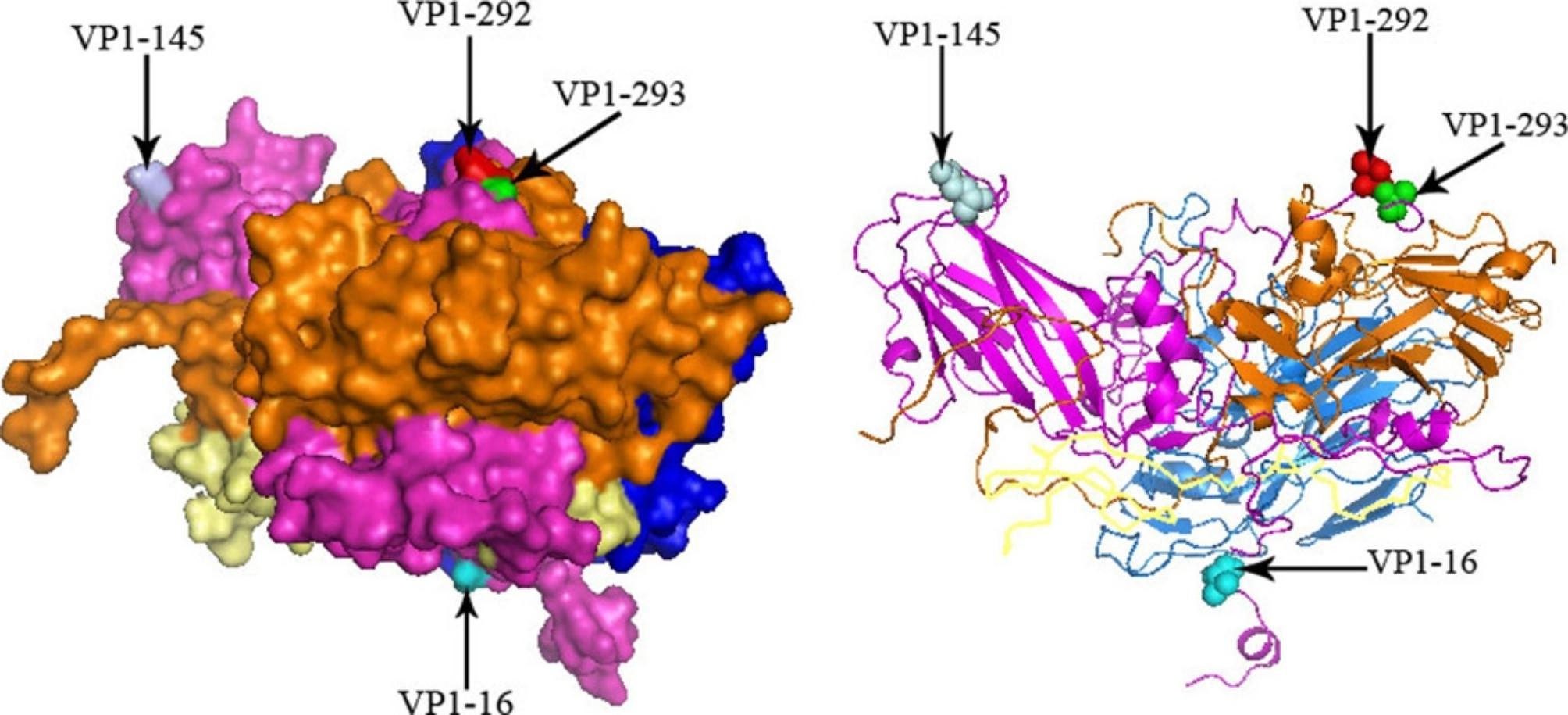
Tertiary structure of the P1 protein estimated with homology model. Four different amino acid residues on the viral capsid protein were labeled on the 3D structure of the EV71 capsid protein using Pymol software. VP1 (purple), VP2 (blue), VP3 (brown), and VP4 (yellow). Sites under positive selection were highlighted as cyan, pale cyan, red, and green, respectively. Letters and numbers refer to the amino acid residues and positions of VP1 in the prototype strain BrCr (U22521.1)

The mixed effects model of evolution (MEME) and single likelihood ancestor counting (SLAC) methods were used to investigate the role of natural selection pressure in EV71 in Beijing (Supplementary Table 6). Individual sites under positive selection were identified. Four key sites (V16M/S, E145Q/G, T292A/K, A293S) were observed from the VP1 sequence dataset. These four amino acid sites were embedded within the loop of the VP1 capsid and were exposed externally (Fig. 3). Two of them, located at amino acids 145 and 293 within VP1 capsid protein, were detected both from the VP1 and P1 data sets (P < 0.05).

### Estimation of Re for EV71

**Fig. 4:**
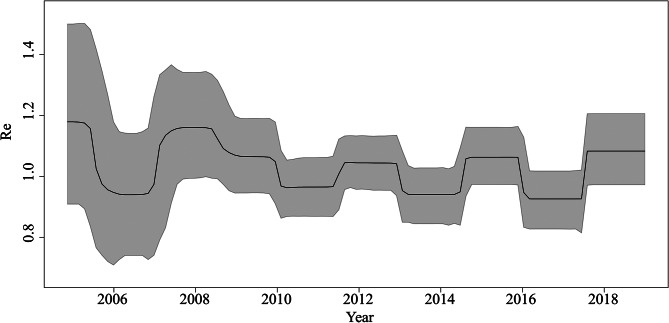
Birth-death skyline plot of the EV71. The curve and the grey area show the mean R_e_ values and their 95% confidence intervals. The y and x-axes represent R_e_ values and time in years, respectively. HPD means the highest posterior density

The analysis showed that the mean Re estimates of EV71 for our dataset were 1.007 (95% HPD, 0.996-1.019), ranged from 0.923 (95% HPD, 0.828‐1.017) to 1.185 (95% HPD, 0.895–1.519) in Beijing. Re estimates of EV71 experienced a typical phylodynamic characterized by a zigzag fluctuation (Fig. 4). The highest Re was observed in 2008 and 2009. It declined after HFMD was listed as one of the class C notifiable infectious diseases in China. The EV71 strains in Beijing originated in an estimated mean of 15.728 years (95%HPD, 14.241‐17.542). The become uninfectious rate was 14.855 (95%HPD, 7.338‐24.737), corresponding to an infectious period of 24.6 (95%HPD, 14.755–49.74) days.

## Discussion

EV71 was the leading pathogen responsible for severe HFMD cases, especially death. During 2009–2019, EV71-associated HFMD decreased greatly in Beijing and in many other provinces and cities [21]. For our consideration, there were two reasons for this declination. The first one was that the pathogen spectrum of HFMD has changed since 2013. Non-CVA16 non-EV71 EV became the major pathogen of HFMD, and EV71-associated HFMD decreased greatly, resulting in less severe cases and fatal cases. Beijing municipality began vaccinating children between 0.5 and 5 years with the EV71 vaccine (self-paid, not compulsory) in August 2016. By the end of 2018, almost 30% of children had received a two-dose vaccine (not published), which was proven highly effective in preventing children from infection [22]. Many children under 5 years of age receiving this vaccine could have reduced the EV71 infection greatly.

Genomic characteristics of EV71 showed that each gene segment shared a high homology among the EV71 sequences in this study, which implied that EV71 prevalent in Beijing shared a common source. 3D region of enterovirus is more susceptible to recombination than any other part [20]. ML phylogenetic trees based on EV71 3D region in Beijing showed that all the 3D sequences clustered together only with lineage I, which was regarded as the dominant type and has never been replaced in China [23]. This result suggested that no recombination events happened in the 3D region from 2009 to 2019 in Beijing.

The relative genetic diversity of the EV71 VP1 sequences first increased and peaked in 2012, which corresponded with the high positive proportion of EV71-associated HFMD in Beijing. With the non-EV71, non-CVA16 EV became the dominant agent, and EV71 genetic diversity declined with fluctuations from 2013 to 2016. After the EV71 vaccine was introduced in China in 2016, genetic diversity declined again. We speculated that it was the vaccine inoculation that prevented children from infection and as a consequence, reduced genetic diversity.

Natural selection pressure analysis showed that the dN/dS value of VP1 and P1 from 2017 to 2019 was smaller than 2009–2016, which implied that both VP1 and P1 had evolved into a relatively stable condition marked by low dN/dS value. Two positive selection sites were obtained from both VP1 and P1 by MEME. VP1-145 is a surface-exposed residue that is located within the DE loop. It has been confirmed that VP1-145 influenced the use of attachment receptors and the three-dimensional structure of the whole virion [24]. Although amino acid residue changed from Ala to Ser on the VP1-293 site has been reported previously [25], further study must confirm whether this correlates with viral pathogenicity.

In this study, the phylogeographic results revealed east China being the primary source sink for the EV71 dispersion all over the country. This is consistent with Zhang’s result [23]. East China is the frontier of reform and opening up and contains many well-developed large cities. They are characterized by frequent international exchanges, active population mobility, and humid and warm climate suitable for the virus survival, which contributes to forming the source of EV71 and plays an important role in disseminating EV71. While for Beijing, the capital of China, a huge migratory population traveling between Beijing and other provinces intensified the spread of the EV71. Therefore, strengthening surveillance of EV71 in Beijing and east China plays an important role in preventing and controlling EV71-associated HFMD.

The epidemic spread of EV71 displayed low Re estimates during the sampling period time, which was close to some previous research [26, 27], suggesting that the introduction of effective prevention and control measures limited viral spread within the sampled populations. The average infectious period in this study agreed with a previous follow-up investigation [28], in which the mild case group turned EV71 negative with a median shedding duration of 18 days, and some cases had a duration of shedding longer than 30 days.

There are limitations to this study. Owing to the severe impact on the surveillance system by the COVID-19 pandemic, the EV71 surveillance result from 2020 to 2022 was not included in this study, which could have provided more information about epidemic characteristics. Not enough EV71 sequences in Beijing or some regions in China of some years might bias the analysis result to a certain extent. Mainly analyzing of VP1 might not be able to clarify the overall epidemic characteristics of EV71, but still help us understand the epidemiological characteristics, phylogenetic features, and bayesian phylodynamics of EV71.

## Conclusion

In conclusion, EV71 was not the major causative agent of HFMD in Beijing in recent years. EV71 prevalent in Beijing from 2009 to 2019, shared high homology in each gene, and were mainly transmitted from east China around 2003. Beijing played an important role in disseminating EV71 to central China. Low Re estimate of EV71 in Beijing implied strategies of prevention and control of HFMD were performed effectively in Beijing.

### Electronic supplementary material

Below is the link to the electronic supplementary material.

Supplementary Material 1

Supplementary Material 2

Supplementary Material 3

Supplementary Material 4

Supplementary Material 5

Supplementary Material 6

Supplementary Material 7

Supplementary Material 8

Supplementary Material 9

Supplementary Material 10

Supplementary Material 11

Supplementary Material 12

## Data Availability

All data and materials described in manuscript are available.

## Abbreviations

EV71: Enterovirus 71
HFMD: Hand, foot and mouth disease
Re: Effective reproductive number
TMRCA: Time to most recent common ancestor
CVA16: Coxsackievirus A16
CVA6: Coxsackievirus A6
NCBI: National Center for Biotechnology Information
BEAST: Bayesian Evolutionary Analysis Sampling Trees
MCMC: Markov chain Monte Carlo
ESS: Effective Sample Size
BSSVS: Bayesian stochastic search variable selection
BF: Bayes factors
BaTS: Bayesian Tip-association Significance Testing
AI: Association Index
PS: Parsimony Score
MC: Monophyletic Clade
COVID-19: Coronavirus disease 2019
ML: Maximum likelihood
