# Correction: Molecular epidemiology and phylodynamic analysis of enterovirus 71 in Beijing, China, 2009–2019

**DOI:** 10.1186/s12985-023-02244-3

**Published:** 2024-01-25

**Authors:** Jie Li, Zhichao Liang, Da Huo, Yang Yang, Renqing Li, Lei Jia, Xiaoli Wang, Chun Huang, Quanyi Wang

**Affiliations:** 1 Institute for HIV/AIDS and STD Prevention and Control, Beijing Center for Disease Prevention and Control, No.16, Hepingli middle Road, Dongcheng District, Beijing, 100013 People’s Republic of China; 2 Institute for Infectious Disease and Endemic Disease Control, Beijing Center for Disease Prevention and Control, No.16, Hepingli middle Road, Dongcheng District, Beijing, 100013 People’s Republic of China; 3 Beijing office of center for global Health, Beijing Center for Disease Prevention and Control, No.16, Hepingli middle Road, Dongcheng District, Beijing, 100013 People’s Republic of China

**Correction: Virology Journal (2023) 20:256**

10.1186/s12985-023-02028-9.

Following publication of the original article [1], the authors informed us about the below errors:

1. Table 2 is repetitively shown.
2. X-axis and Y-axis are missing in Fig. 4.
3. The supplementary materials 1, 7, 8, 9, 10, 11 and 12 need to be updated.

The original article has been corrected.
