# Supplementary material for: Molecular epidemiology and phylodynamic analysis of enterovirus 71 in Beijing, China, 2009–2019
Source: Virol J. 2023 Nov 3;20:256. doi: 10.1186/s12985-023-02028-9 (PMC10625277; doi:10.1186/s12985-023-02028-9)
Supplement: Supplementary file 1 — Supplementary Material 1 [file 12985_2023_2028_MOESM1_ESM.docx]

Supplementary Figure 1:

Reported case number of EV71-associated HFMD and incidence rate of HFMD in Beijing, China, 2009-2019.

Supplementary Figure 2:

Severe case number and fatal case number of EV71 causative HFMD in Beijing, China, 2009-2019.

Supplementary Figure 3:

Yearly distribution of enterovirus serotypes in laboratory-confirmed HFMD cases in Beijing, during 2009 and 2019.

Supplementary Figure 4:

Maximum likelihood phylogenetic trees of genes from EV71 strains. Phylogenetic trees were constructed using the VP4, VP2, VP3, VP1, 2A, 2B, 2C, 3AB, 3C and 3D coding region of the 86 EV71 genomes with prototype strains from HEV-A as the references.

Supplementary Figure 5:

Maximum likelihood phylogenetic tree of 3D coding region. Maximum likelihood phylogenetic tree was constructed using the 3D coding region of 86 EV71 genomes with strains from 17 evolutionary lineages (A, B, C, D, E, F, G, H, I, J, K, L, M, N, O, P, and Q) as the references. Each lineage was coded with a different color. Sequences of 3D coding region in this study were all clustered in lineage I.
