# Supplementary material for: Molecular epidemiology and phylodynamic analysis of enterovirus 71 in Beijing, China, 2009–2019
Source: Virol J. 2023 Nov 3;20:256. doi: 10.1186/s12985-023-02028-9 (PMC10625277; doi:10.1186/s12985-023-02028-9)
Supplement: Supplementary file 7 — Supplementary Material 7 [file 12985_2023_2028_MOESM7_ESM.docx]

Supplementary Table 1. EV, EV71, CVA16 and CVA6 were identified with real-time RT-PCR kits

| Detection Kit | Target pathogen | PCR cycling conditions |
| --- | --- | --- |
| 1. EV71, CVA16 and EV nucleic acid detection kit by triple fluorescent real-time RT-PCR | EV71, CVA16, and EV | 50 °C for 15 min, 95 °C for 15 min, followed by 40 cycles of 94 °C for 15 sec, 55 °C for 45 sec. |
| 1. CVA6 nucleic acid detection kit by real-time RT-PCR | CVA6 | 50 °C for 15 min, 95 °C for 15 min, followed by 40 cycles of 94 °C for 15 sec, 55 °C for 45 sec. |
