# Supplementary material for: Molecular epidemiology and phylodynamic analysis of enterovirus 71 in Beijing, China, 2009–2019
Source: Virol J. 2023 Nov 3;20:256. doi: 10.1186/s12985-023-02028-9 (PMC10625277; doi:10.1186/s12985-023-02028-9)
Supplement: Supplementary file 8 — Supplementary Material 8 [file 12985_2023_2028_MOESM8_ESM.docx]

Supplementary Table 2. The information of 86 enterovirus A71 (EV71) strains used in this analysis, including 79 isolates first reported in this study

| Strain name | Isolation year | Type of specimen | Source | *GenBank accession No. |
| --- | --- | --- | --- | --- |
| 2019SD7_8/Beijing/China | 2019 | Throat swab | This study | OQ355732 |
| 2019SY55_1/Beijing/China | 2019 | Throat swab | This study | OQ355733 |
| 2019SY8_1/Beijing/China | 2019 | Throat swab | This study | OQ355734 |
| 2019SY38_3/Beijing/China | 2019 | Throat swab | This study | OQ355735 |
| 2019SY35_12/Beijing/China | 2019 | Throat swab | This study | OQ355736 |
| 2019SD7_9/Beijing/China | 2019 | Throat swab | This study | OQ355737 |
| 2019SY51_9/Beijing/China | 2019 | Throat swab | This study | OQ355738 |
| 13/BJ/CHN/2018/Beijing/China | 2018 | Throat swab | Beijing | ON502189 |
| 9/BJ/CHN/2018/Beijing/China | 2018 | Throat swab | Beijing | ON502291 |
| 20/BJ/CHN/2018/Beijing/China | 2018 | Throat swab | Beijing | ON502213 |
| 14/BJ/CHN/2018/Beijing/China | 2018 | Throat swab | Beijing | ON502194 |
| 19/BJ/CHN/2018/Beijing/China | 2018 | Throat swab | Beijing | ON502208 |
| 2018SH58_7/Beijing/China | 2018 | Throat swab | This study | OQ355739 |
| 2018SH44_3/Beijing/China | 2018 | Throat swab | This study | OQ355740 |
| 2018SH1_3/Beijing/China | 2018 | Throat swab | This study | OQ355741 |
| 2018SH38_4/Beijing/China | 2018 | Throat swab | This study | OQ355742 |
| 2018SH35_5/Beijing/China | 2018 | Throat swab | This study | OQ355743 |
| 2018SH9_2/Beijing/China | 2018 | Throat swab | This study | OQ355744 |
| 2018SH57_14/Beijing/China | 2018 | Throat swab | This study | OQ355745 |
| 2018SH53_46/Beijing/China | 2018 | Throat swab | This study | OQ355746 |
| 2018SH44_9/Beijing/China | 2018 | Throat swab | This study | OQ355747 |
| 2018SH25_7/Beijing/China | 2018 | Throat swab | This study | OQ355748 |
| 2017SH28_6/Beijing/China | 2017 | Throat swab | This study | OQ355749 |
| 2017SH85_4/Beijing/China | 2017 | Throat swab | This study | OQ355750 |
| 2017SH65_11/Beijing/China | 2017 | Throat swab | This study | OQ355751 |
| 2017SH84_55/Beijing/China | 2017 | Throat swab | This study | OQ355752 |
| 2017SH82_8/Beijing/China | 2017 | Throat swab | This study | OQ355753 |
| 2017SH77_14/Beijing/China | 2017 | Throat swab | This study | OQ355754 |
| 17/BJ/CHN/2017/Beijing/China | 2017 | Throat swab | Beijing | ON502201 |
| 22/BJ/CHN/2017/Beijing/China | 2017 | Throat swab | Beijing | ON502218 |
| 2017SH85_2/Beijing/China | 2017 | Throat swab | This study | OQ355755 |
| 2016sy28_17/Beijing/China | 2016 | Throat swab | This study | OQ355756 |
| 2016SY67_12/Beijing/China | 2016 | Throat swab | This study | OQ355757 |
| 2016sy44_7/Beijing/China | 2016 | Throat swab | This study | OQ355758 |
| 2016sy68_1/Beijing/China | 2016 | Throat swab | This study | OQ355759 |
| 2016SD6_8/Beijing/China | 2016 | Throat swab | This study | OQ355760 |
| 2016sy67_10/Beijing/China | 2016 | Throat swab | This study | OQ355761 |
| 2016sy28_14/Beijing/China | 2016 | Throat swab | This study | OQ355762 |
| 2015sy63_60/Beijing/China | 2015 | Throat swab | This study | OQ355763 |
| 2015sy85_3/Beijing/China | 2015 | Throat swab | This study | OQ355764 |
| 2015sy51_3/Beijing/China | 2015 | Throat swab | This study | OQ355765 |
| 2015sy34_5/Beijing/China | 2015 | Throat swab | This study | OQ355766 |
| 2015sy73_1/Beijing/China | 2015 | Throat swab | This study | OQ355767 |
| 2015sy73_9/Beijing/China | 2015 | Throat swab | This study | OQ355768 |
| 2015sy12_1/Beijing/China | 2015 | Throat swab | This study | OQ355769 |
| 2015sy5_2/Beijing/China | 2015 | Throat swab | This study | OQ355770 |
| 2015sy112_1/Beijing/China | 2015 | Throat swab | This study | OQ355771 |
| 2015sy83_31/Beijing/China | 2015 | Throat swab | This study | OQ355772 |
| 2015sy116_9/Beijing/China | 2015 | Throat swab | This study | OQ355773 |
| 2014SY42_11/Beijing/China | 2014 | Throat swab | This study | OQ355774 |
| 2014SY29_1/Beijing/China | 2014 | Throat swab | This study | OQ355775 |
| 2014SY27_3/Beijing/China | 2014 | Throat swab | This study | OQ355776 |
| 2014SY47_69/Beijing/China | 2014 | Throat swab | This study | OQ355777 |
| 2014SY32_6/Beijing/China | 2014 | Throat swab | This study | OQ355778 |
| 2014SY57_6/Beijing/China | 2014 | Throat swab | This study | OQ355779 |
| 2014SY27_2/Beijing/China | 2014 | Throat swab | This study | OQ355780 |
| 2014SY45_11/Beijing/China | 2014 | Throat swab | This study | OQ355781 |
| 2013SY62_2/Beijing/China | 2013 | Throat swab | This study | OQ355782 |
| 2013SY72_57/Beijing/China | 2013 | Throat swab | This study | OQ355783 |
| 2013SY132_2/Beijing/China | 2013 | Throat swab | This study | OQ355784 |
| 2013SY72_23/Beijing/China | 2013 | Throat swab | This study | OQ355785 |
| 2013SY72_55/Beijing/China | 2013 | Throat swab | This study | OQ355786 |
| 2013SY65_6/Beijing/China | 2013 | Throat swab | This study | OQ355787 |
| 2013SY59_10/Beijing/China | 2013 | Throat swab | This study | OQ355788 |
| 2013SY72_16/Beijing/China | 2013 | Throat swab | This study | OQ355789 |
| 2013SY62_4/Beijing/China | 2013 | Throat swab | This study | OQ355790 |
| 2012SY25_43/Beijing/China | 2012 | Throat swab | This study | OQ355791 |
| 2012SY14_2/Beijing/China | 2012 | Throat swab | This study | OQ355792 |
| 2012SY20_1/Beijing/China | 2012 | Throat swab | This study | OQ355793 |
| 2012SY16_15/Beijing/China | 2012 | Throat swab | This study | OQ355794 |
| 2012SY21_11/Beijing/China | 2012 | Throat swab | This study | OQ355795 |
| 2011HD014/Beijing/China | 2011 | Throat swab | This study | OQ355796 |
| 2011HD023/Beijing/China | 2011 | Throat swab | This study | OQ355797 |
| 2011HD012_1/Beijing/China | 2011 | Throat swab | This study | OQ355798 |
| 2011HD146/Beijing/China | 2011 | Throat swab | This study | OQ355799 |
| 2011HD099/Beijing/China | 2011 | Throat swab | This study | OQ355800 |
| 2010SD7_5/Beijing/China | 2010 | Throat swab | This study | OQ355801 |
| 2010SD7_1/Beijing/China | 2010 | Throat swab | This study | OQ355802 |
| 2010HY_265/Beijing/China | 2010 | Throat swab | This study | OQ355803 |
| 2010SY32_1/Beijing/China | 2010 | Throat swab | This study | OQ355804 |
| 2010HY_380/Beijing/China | 2010 | Throat swab | This study | OQ355805 |
| 2009_512/Beijing/China | 2009 | Throat swab | This study | OQ355806 |
| 2009_812/Beijing/China | 2009 | Throat swab | This study | OQ355807 |
| 2009_1097/Beijing/China | 2009 | Throat swab | This study | OQ355808 |
| 2009_34/Beijing/China | 2009 | Throat swab | This study | OQ355809 |
| 2009_269/Beijing/China | 2009 | Throat swab | This study | OQ355810 |
