# Supplementary material for: Molecular epidemiology and phylodynamic analysis of enterovirus 71 in Beijing, China, 2009–2019
Source: Virol J. 2023 Nov 3;20:256. doi: 10.1186/s12985-023-02028-9 (PMC10625277; doi:10.1186/s12985-023-02028-9)
Supplement: Supplementary file 9 — Supplementary Material 9 [file 12985_2023_2028_MOESM9_ESM.docx]

Supplementary Table 3. Geographic and temporal information of the EV71 VP1 sequences used in this study.

| Year | Beijing | Central | East | North | South | West | Total |
| --- | --- | --- | --- | --- | --- | --- | --- |
| 2006 | 1 | 0 | 1 | 0 | 0 | 0 | 2 |
| 2007 | 2 | 0 | 3 | 2 | 0 | 0 | 7 |
| 2008 | 3 | 0 | 10 | 2 | 3 | 4 | 22 |
| 2009 | 3 | 4 | 8 | 0 | 5 | 1 | 21 |
| 2010 | 9 | 5 | 8 | 1 | 6 | 4 | 33 |
| 2011 | 3 | 4 | 6 | 0 | 4 | 0 | 17 |
| 2012 | 6 | 4 | 5 | 0 | 3 | 0 | 18 |
| 2013 | 8 | 1 | 7 | 0 | 4 | 0 | 20 |
| 2014 | 8 | 1 | 9 | 0 | 3 | 2 | 23 |
| 2015 | 8 | 1 | 7 | 0 | 2 | 1 | 19 |
| 2016 | 6 | 7 | 12 | 7 | 17 | 14 | 63 |
| 2017 | 7 | 13 | 8 | 5 | 9 | 6 | 48 |
| 2018 | 7 | 10 | 1 | 8 | 10 | 9 | 45 |
| 2019 | 5 | 1 | 1 | 0 | 5 | 3 | 15 |
| Total | 76 | 51 | 86 | 25 | 71 | 44 | 353 |
