# Supplementary material for: Molecular epidemiology and phylodynamic analysis of enterovirus 71 in Beijing, China, 2009–2019
Source: Virol J. 2023 Nov 3;20:256. doi: 10.1186/s12985-023-02028-9 (PMC10625277; doi:10.1186/s12985-023-02028-9)
Supplement: Supplementary file 10 — Supplementary Material 10 [file 12985_2023_2028_MOESM10_ESM.docx]

Supplementary Table 4: The nucleotide divergence of overall mean distance of each gene among EV71 strains in Beijing, China, 2009-2019

| Gene | P1 | | | |  | P2 | | |  | P3 | | | | | |
| --- | --- | --- | --- | --- | --- | --- | --- | --- | --- | --- | --- | --- | --- | --- | --- |
|  | VP4 | VP2 | VP3 | VP1 |  | 2A | 2B | 2C |  | 3A | 3B | 3C | 3D | |  |
| Mean distance | 0.043 | 0.049 | 0.054 | 0.051 |  | 0.052 | 0.066 | 0.049 |  | 0.060 | 0.089 | 0.042 | | 0.051 | |
