# Supplementary material for: Molecular epidemiology and phylodynamic analysis of enterovirus 71 in Beijing, China, 2009–2019
Source: Virol J. 2023 Nov 3;20:256. doi: 10.1186/s12985-023-02028-9 (PMC10625277; doi:10.1186/s12985-023-02028-9)
Supplement: Supplementary file 11 — Supplementary Material 11 [file 12985_2023_2028_MOESM11_ESM.docx]

Supplementary Table 5: Analysis of the geography structure of the EV71 strains in China

| Statistic | Isolates | Observed mean(95%HPD) | Null mean(95%HPD) | Significance |
| --- | --- | --- | --- | --- |
| AI |  | 22.26(20.71, 23.83) | 30.42(28.92, 31.70) | <0.001*** |
| PS |  | 171.82(167.00, 176.00) | 216.99(210.02, 223.10) | <0.001*** |
| MC (Beijing) | 76 | 4.01(3.00, 6.00) | 1.50(1.00, 2.01) | 0.009999990** |
| MC (Central) | 51 | 4.00(4.00, 4.00) | 1.86(1.21, 2.44) | 0.009999990** |
| MC (East) | 86 | 2.62(2.00, 3.00) | 2.38(2.01, 3.09) | 0.110000014 |
| MC (North) | 25 | 2.45(2.00, 3.00) | 2.78(2.12, 4.04) | 1.000000000 |
| MC (South) | 71 | 5.83(3.00, 9.00) | 2.40(2.00, 3.15) | 0.009999990** |
| MC (West) | 44 | 2.93(2.00, 4.00) | 2.03(1.40, 2.92) | 0.040000021* |

AI, association index; PS, parsimony score; MC, maximum monophyletic clade; HPD, highest probability density interval. Significance thresholds: *0.01 < P<0.05; **0.001 < P<0.01; ***P<0.001.
