# Supplementary material for: Molecular epidemiology and phylodynamic analysis of enterovirus 71 in Beijing, China, 2009–2019
Source: Virol J. 2023 Nov 3;20:256. doi: 10.1186/s12985-023-02028-9 (PMC10625277; doi:10.1186/s12985-023-02028-9)
Supplement: Supplementary file 12 — Supplementary Material 12 [file 12985_2023_2028_MOESM12_ESM.docx]

Supplementary Table 6: Natural selection pressures for VP1 and P1 region of EV71 from different data sets

| Year |  | VP1 | | |  | P1 | | |
| --- | --- | --- | --- | --- | --- | --- | --- | --- |
|  |  | Datasets | dN/dS(SLAC) | Positively selection sites by MEME* |  | Datasets | dN/dS(SLAC) | Positively selection sites by MEME* |
| 2009-2019 |  | 156 | 0.0499 | 16, 145, 292 293 |  | 86 | 0.0240 | 145, 293 |
| 2009-2016 |  | 114 | 0.0534 | 16, 145, 292 293 |  | 55 | 0.0247 | 145, 293 |
| 2017-2019 |  | 42 | 0.0307 | 145 |  | 31 | 0.0191 | 145 |

*The numbers refer to the amino acid positions of VP1 protein in the prototype strain BrCr (U22521.1)
